# Experimentally induced changes in authoritarian submission as a response to threat

**DOI:** 10.1038/s41598-023-44713-3

**Published:** 2023-10-31

**Authors:** Taylor Winter, Benjamin C. Riordan, Damian Scarf, Paul E. Jose

**Affiliations:** 1 School of Psychology, Victoria University of Wellington, Wellington, New Zealand; 2 School of Mathematics and Statistics, University of Canterbury, Christchurch, New Zealand; 3 Centre for Alcohol Policy Research, La Trobe University, Melbourne, Australia; 4 Department of Psychology, University of Otago, Dunedin, New Zealand

**Keywords:** Psychology, Statistics

## Abstract

Authoritarianism is best conceptualised by three attitudinal clusters: Aggression, Submission, and Conventionalism. Once considered a fixed characteristic, recent observational research has demonstrated how the dimension of submission can fluctuate in response to COVID-19 threat as a means of maintaining collective security. However, this effect has not been investigated with other forms of threat, nor has it been supported experimentally. In the present study, we sought to test observational findings by priming 300 participants with either a COVID-19 threat, a domestic terrorism threat, or a non-threatening control. Levels of authoritarianism were tested before and after presentation of a prime and then the difference between the two measures could be compared between prime conditions. Results from a Bayesian multivariate regression analysis informed by observational findings suggested that participants who experienced the COVID-19 or terrorism primes reported higher levels of authoritarian submission after the prime compared to before the prime, relative to those who experienced the neutral control prime. In contrast, the aggression subfactor did not seem to elicit any change in response to threat, and the conventionalism subfactor showed a response only to the terrorism prime. We concluded that two different forms of societal threat could elicit changes in specific dimensions of authoritarianism over a very short time span. We caution against the common practice of treating authoritarianism as a unidimensional construct without careful consideration.

## Introduction

Research on the nature of authoritarianism has made significant progress over the last decade. Initially treated as a fixed personality trait, it is now generally accepted that authoritarianism is a state which can fluctuate due to context^1^. Moreover, it is increasingly evident that authoritarianism is not exclusively a politically right-wing phenomenon^2^. These new developments have led to more questions than answers as our models of authoritarianism grow in complexity. We have established that elements of authoritarianism can fluctuate over time and are associated with changes in perceived threats (e.g., pandemics, natural disasters^2–4^). A limitation of past research is the lack of experimental designs used to investigate authoritarianism and related constructs. To address this issue, the current study uses an experimental approach to investigate which aspects of authoritarianism fluctuate in response to threat.

Authoritarianism is conventionally comprised of three attitudinal clusters: *Aggression* to those individuals who oppose conformity and leadership of the group; *submission* to the group leader; and adherence to *conventional* traditions and values of the group^5–7^. (Although we follow Duckitt et al.’s^6^ ACT model, we retain the naming convention for dimensions used by Altemeyer^5^. This is because the equivalent of the submission dimension is referred to as the conservatism dimension which can cause confusion when also investigating political conservatism.) This tripartite conceptualisation of authoritarianism is consistent across the two leading contemporary theorists, Altemeyer and Duckitt, but where they differ is that Duckitt posited that dimensions could fluctuate in response to threat. When a threat is introduced to the group, the type of threat, and the reception to addressing the threat by both in-group and out-group members, determines which dimensions fluctuate and to what extent. The underlying function of fluctuations in authoritarian dimensions are because threat evokes dangerous world views that people are motivated to address through social cohesion. Each dimension can increase social cohesion in slightly different ways, with submission being the most obvious as all in-group members will follow the guidance of an authoritarian leader(s). Aggression is reserved for dissenters or out-group members that seek to disrupt social cohesion, and conventionalism is a common set of rules or beliefs that all members diligently follow. But is an isolated increase in submission possible given that all dimensions of authoritarianism are so highly correlated? After the model’s initial proposal^6,8,9^, research has demonstrated that the factors are indeed independent to varying extents, such that changes in one factor may occur without changes in the other two^2,10,11^.

In one recent example, Winter et al.^2^ conducted a cross-sectional survey of 1430 community adults during the highest level of lockdown for the COVID-19 pandemic in New Zealand. They believed from analysis of media discourse, that the New Zealand population was demonstrating high levels of authoritarian submission towards Prime Minister Jacinda Ardern and the Director-General of Health, Ashley Bloomfield. They demonstrated that levels of authoritarian submission were similar between individuals on the political left- and right-wings while, consistent with previous literature, aggression and conventionalism were higher for those individuals on the political right^6^. It was proposed that the similarity between left- and right-wing participants on the submission subscale was attributable to the societal threat posed by the contemporaneous pandemic. Supporting this view, Winter et al.^4^ collected a second wave of data from the same sample and demonstrated longitudinally that, when the threat of COVID-19 had dissipated, levels of authoritarian submission decreased. However, even this work has its limitations. Although informative, cross-sectional and longitudinal data cannot speak directly to the causal factors driving changes in authoritarian submission. Consequently, to identify a specific mechanism that could explain changes in components of authoritarianism, experimental studies are needed.

One particular concern with longitudinal observational studies is that threat is not randomly assigned across the population, and confounding variables can determine to what extent an individual perceives a threat. Thus, change in authoritarian attitudes may differ based on confounds rather than the direct influence of a threat. There is also a limitation when utilising COVID-19 as a threat, in that carefully following leadership during a pandemic is sound science-based decision making rather than a pure exhibition of authoritarian submission. Experimental studies allow us to control who experiences a threat, the severity of the threat, and the type of threat to compare whether pandemic responses in authoritarianism carry over to other forms of threat.

Experimental studies that have attempted to manipulate levels of authoritarianism are few, let alone studies that directly assess the impact of global threats like a global pandemic^3,12–14^. Concerning localised threats, Russo et al.^3^ presented participants with a single vignette that manipulated threat levels using regional crime rates. Participants who perceived a high level of threat displayed an increase in authoritarianism relative to those who perceived a low level of threat.

To date, experimental studies have treated authoritarianism as a unidimensional construct^3^. As we note above, longitudinal work suggests that specific dimensions of authoritarianism (e.g., submission) can fluctuate in response to changes in threat. Building on this finding, in the current study, we assessed the impact of threats on authoritarian aggression, submission, and conventionalism^6^. We primed participants with one of two threats (COVID-19 pandemic and domestic terrorism). The use of two distinctly different threats allow us to test whether the now well supported change in authoritarian submission during the COVID-19 pandemic carries over to other forms of threat. Using domestic terrorism was a logical choice from previous studies that had investigated elicitation of authoritarian unidimensionally, and the threat is also categorically different to a pandemic.

All three authoritarian subfactors were tested before and after being primed with a one of the two threats or a neutral control prime. We hypothesised that participants in both the COVID-19 and terrorism prime conditions would report higher levels of authoritarian submission after the prime compared to before the prime, relative to any change observed in the neutral control condition. Importantly, our hypotheses stipulated that fluctuations in submission induced by perceived threat are generalisable to a wide range of threats and are not unique to COVID-19 contexts. Further, we hypothesised that aggression and conventionalism would remain the same before and after the prime, i.e., not exhibit responsiveness to threat. The hypothesis regarding submission being the only dimension that changes, is aligned with previous research on media discourse during the COVID-19 pandemic, and on both a cross-sectional and longitudinal observation^2,4^. These previous studies sit well with the motivational model of authoritarianism that suggested submission can change independently of the two other dimensions in response to threat as a form of society-wide coping mechanism to protect against a dangerous world.

## Methods

### Participants

We recruited 301 participants from the United States using the online survey platform Amazon Mechanical Turk. The sample size was determined as a factor of sample sizes in observational research, expected influence of Bayesian priors, and funding available to pay participants. Participants were paid USD2.50 for completing the 15-min survey and participation was limited to ‘Masters’ workers who have a proven track record of high quality engagement as determined by Amazon. Due to the random assignment of participants to experimental conditions, we obtained slightly fewer participants in the COVID-19 condition and fewer females overall (see Table 1). Multinomial Bayesian regression was used to determine if there were any differences among the participants allocated to the three conditions, indicating significantly higher political beliefs in the terror prime relative to control, with a posterior probability (pp) of 96%. We also noted that participants in the COVID-19 prime were significantly older than in the control prime (pp = 0.99). No other statistically significant effects of age, sex, or political beliefs were noted between the three groups (pp < 0.95). We observed heavy overlap among the three conditions’ distributions and we did not expect these minor demographic differences to bias or obscure the proposed analyses (which included demographic covariates as a precaution). Thus, we concluded that groups were representative and similar across age, sex, and political beliefs (assessed with a Likert scale ranging 1 to 7; see Table 1). Three participants preferred not to declare their biological sex and/or age and were removed from subsequent analysis. Data was collected on the 24^th^ of September 2021, four days after the death toll surpassed that of the Spanish Flu. The delta variant had become the predominant strain two months prior and there were early reports of the new Omicron strain overseas. Ethical approval was granted by the University of Otago Ethics Committee. The primes involving threats were intended to be transitory, and debriefing ensured that all participants returned to a baseline level of concern at the conclusion of the study.

**Table 1:** Participant demographics for each experimental condition.

Condition	Count	Male (%)	Age		Political beliefs	
			M [SD]	Range	M	SD
Control	107	58%	40.9 [11.45]	23–89	4.2	1.78
COVID-19	88	65%	44.3 [10.71]	27–74	4.4	1.90
Terrorism	103	49%	43.4 [10.92]	25–77	4.7	1.93

Political beliefs is a Likert scale ranging from 1—totally liberal to 7—totally conservative.

### Procedure

The survey was conducted in late September 2021, during the Biden presidency in the United States. Mask mandates and other restrictions had been gradually lifting in various geographical regions, but at this point there had been more cases in the United States than reported during the Spanish flu (43 million) and 1 in 500 Americans had died of COVID-19. Upon initiating the survey, participants were presented with an information sheet concerning the study, and they provided informed consent if they decided to continue. Participants were randomly assigned to a single experimental condition (between-subjects design) consisting of a prime constituted by a short paragraph and digitally presented newspaper clippings on either domestic terrorism, COVID-19, or a neutral control. All participants then completed a COVID-19 threat scale and a domestic terrorism threat scale to test the experimental manipulation. Authoritarianism was tested before and after the prime to test our hypothesis that threat would elicit a transient increase in submission but not aggression or conventionalism.

### Planned missing data design

Authoritarianism and its subfactors were measured using the ACT scale consisting of 36 Likert scales^6^. We randomly assigned six out of 12 questions from each of the three ACT subscales and presented them before the prime, with the remaining six questions for each subscale being presented after the prime (so participants never saw the same question twice). This planned missing data design (Little and Rhemtulla 2013^15^; Rhemtulla and Little 2012^16^) reduces respondent burden and pre-test sensitisation (i.e., participants answer 36 fewer questions and only experience each question once in the survey). Planned missing data designs leverage the fact that data are missing completely at random (MCAR) because questions are selected randomly by the survey tool Qualtrics, allowing non-biased imputation because missingness is random.

### Primes

Each prime consisted of a blurb and, in the case of domestic terrorism and COVID-19, a collage of recent news clippings. To ensure participants engaged with news clippings, they were asked to indicate which clipping they viewed as the most concerning. Each blurb is presented below, and the news clipping collages are presented in Appendix A. Note that COVID-19 is mentioned across all three primes. This was due to the high COVID-19 threat environment in which this experiment was conducted. We attempted to quell the threat of COVID-19 in other conditions.

#### COVID-19

*“There are many threats to us as individuals and society more broadly. However, the most considerable, and impactful threat is the current COVID-19 environment. There have been continuing challenges such as the threat of new variants (Delta and Mu) that are more contagious or vaccine resistant (See examples of recent headlines below). It is therefore important now more than ever that we understand the public’s views on leadership and authority, to best respond to future economic and health threats due to COVID-19.”*

#### Domestic terrorism

*“There are many types of threat to us as individuals and society more broadly. Currently there is the COVID-19 pandemic as a threat, but another type of threat is terrorism. A growing threat both in the US and globally is due to domestic terrorists and overseas terrorists. Terrorist attacks have been growing in prevalence both physically, e.g., bombings, and computer-based, e.g., stealing social security information, or cyberattacks on large infrastructure such as oil pipelines using ransomware (see examples of recent headlines below). It is therefore important now more than ever that we understand the public’s views on leadership and authority, to best respond to future terrorist threats, however they may present themselves.”*

#### Control (neutral)

*“In recent times, there has been large changes in our beliefs and views that govern our everyday decision making. It is important to understand how the changing population views leadership and authority as our environment continues to change. In this vein, it is important to understand what threats you perceive in society, be it from COVID-19, violence, or other threats. For example, COVID-19 is increasingly becoming better managed, and the risks are decreasing day by day within the United States. However, it is still something that will take time to recover from and may indirectly impact your views and beliefs.”*

### Measures

#### Political beliefs

We asked participants their political beliefs using a seven-point Likert scale ranging from 1—*Totally conservative* to 4—*Moderate* to 7—*Totally liberal*. This single item has been used in our previous research to understand how peoples attitudes and behaviours can change as a function of political orientation, where liberal and conservative is synonymous with left- and right-wing orientation.

#### COVID-19 threat scale

The perceived threat of COVID-19 was measured using a ten-item self-report scale^17^. The scale starts with the prompt ‘On March 11th 2020 the World Health Organization declared COVID-19, a viral disease that has swept the globe, a pandemic. How much of a threat, if any, is the coronavirus outbreak for…’. Example items are ‘*Your personal health*’ and ‘*The U.S. economy*’ with responses collected on a 4-point Likert scale ranging from 1—*Not a threat* to 4—*Major threat*. The scale was developed to separately measure both realistic and symbolic threats to sociocultural identity as they predict behavioural responses to COVID-19 policies. The scale was validated across three samples and has been widely used in understanding pandemic related behaviour change.

#### Terrorism catastrophising scale

A shortened scale of terrorism catastrophising was used to capture fear of terrorism^18^. The original scale is 13 items and we used 9 of the best-performing items from the scale (see Appendix B). Responses were recorded using a 5-point Likert scale ranging from 1—*Strongly disagree* to 5—*Strongly agree*, with examples such as ‘*I often dwell on the future threat of terrorism*’. The scale was originally developed for measuring catastrophising in the context of terror management theory and cognitive-behavioural theory. It is built off of three dimensions being rumination, magnification, and helplessness in the face of a terrorist threat. Although not widely used, it has undergone full validation and confirmatory analysis yielded a CFI of 0.96 in the original study on an internet based US population.

#### Aggression–conservatism–traditionalism (ACT) scale

Authoritarianism was measured using the ACT scale, consisting of six positively worded items and six negatively worded items for each subscale, for a total of 36 items^6^. The authoritarian aggression subfactor consisted of questions such as ‘*Our government does NOT need tougher government and stricter laws*’ (reverse-scored). The submission subfactor consisted of questions such as ‘*It’s great that many young people today are prepared to defy authority*’ (reverse-scored). Lastly, the conventionalism subfactor included questions such as ‘*It is important that we preserve our traditional values and moral standards*’. All responses were given on a 7-point Likert scale ranging from 1—*Strongly disagree* to 7—*Strongly agree*. This scale is well validated in the literature and has been in constant use for over a decade. It has strong ties to the RWA scale developed by Bob Altemeyer, but instead takes a more neutral approach of general authoritarianism rather than taking a specific right-wing position.

### Analytical approach

#### Multiple imputation

The ACT scale responses that were randomly missing by design were imputed using Multiple Imputation by Chained Equations (MICE) using the *mice* package in R programming language^19,20^. MICE was used because it allowed us to sample a range of likely imputed values to give an overview of imputation uncertainty^21^. One drawback is that MICE introduces some random noise in the estimation process called simulation error. However, the simulation error is reduced as the number of imputed datasets increases. In Eq. (1), we express the relationship between the estimated variance without simulation error (T_∞_) relative to the variance with simulation error given for *m* number of imputed datasets (T_m_). In other words, a dataset with an infinite number of imputations experiences no simulation error, and for *m* number of datasets, simulation error will increase variance by a factor of (1 + Y_0_ * m) where y_0_ is the percentage of missing data for a given variable. If we use the common rule of thumb of 20 imputed datasets, then with 50% missing data for the ACT scale we would approximate simulation error to inflate variance by (1 + 0.5 * 20) or a 1.025 times increase in variance. We therefore decided to use 50 imputed datasets to minimize error (giving a coefficient of 0.01). Age, sex, political beliefs, and condition were entered alongside all ACT scale questions for imputation, however, the pre-prime and post-prime questions were all imputed and analysed in separate analyses so they did not bias one another. We could also expect clustering within each prime condition, and therefore added a random effect for the prime when imputing post-prime questions.

Relationship between estimated variance and estimated variance with simulation error as discussed by Buuren and Groothuis-Oudshoorn^19^.

1 $${T}_{m}=\left(1+ \frac{{\gamma }_{0}}{m}\right)\cdot {T}_{\infty }$$

#### Latent constructs

Scale measures were summarised using Confirmatory Factor Analysis (CFA) to provide latent measures implemented through the *lavaan* package^22^. Multiply imputed datasets were estimated and summarised automatically during CFA using the *semTools* package^23^. We determined adequate fit of models based on a CFI of more than 0.95 and RMSEA below 0.08. The ACT scale was modelled with all three subfactors separately feeding into a single higher-order ACT scale factor. The *plausibleValues* R function was used to generate 50 draws from each imputed dataset and the mean was taken to estimate each latent variable.

#### Manipulation check

A Bayesian multivariate regression was used to test whether terrorism and COVID-19 primes elicited significantly higher levels of threat relative to the control prime. In each of two regression analyses, the prime condition, age, sex, and political beliefs were entered as covariates with either the COVID-19 threat, or terrorism catastrophizing measure as the outcome. We used default priors for this Bayesian analysis.

#### Testing for change in authoritarianism

A multivariate Bayesian regression tested whether each authoritarian subfactor increased after exposure to either the COVID-19 or terrorism prime relative to the control prime. Each subfactor after exposure was an outcome, with age, sex, political beliefs, and condition as predictors. We entered each subfactor’s baseline, or pre-exposure, level to form a multivariate lagged regression (Eq. 2). We used informative priors on all predictors, and non-informative analyses are included in the supplementary information.

Lagged regression model for each of the three subfactors (note that the covariances between outcomes is omitted).

2 $${Subfactor}_{Post} \sim {Subfactor}_{Pre}+Condition+Age+Sex+Political \; beliefs$$

#### Summary statistics in our Bayesian analyses

In the present paper we use a Bayesian framework to conduct regression analysis. This approach uses two similar yet distinctly different concepts to frequentist analysis of which readers may be unaware, and we explain them here. Firstly instead of 95% confidence intervals we report the 95% credible interval, which is the band within which the true value of a statistic lies with 95% probability. We derive this statistic by taking the posterior distribution, which is a collection of estimates, and calculate the 2.5th and 97.5th percentiles, a definition of the 95% CI called the Equal Tailed Interval (ETI). We also determine whether an effect is meaningfully different to zero by using a Posterior Probability (pp) which is functionally like a p-value. The pp is relative and has no specific definition so attention must be given to how authors choose to apply it. In the present paper, we calculate the pp of an effect being greater, or less than zero. That is, if 80% of posterior samples fall above zero then the pp is 80%. If 20% of the posterior samples fall above zero, then the pp is also 80% because naturally 80% of the posterior samples must be below zero. Therefore, the pp is functionally similar to the p-value and you cannot determine the direction of the effect without knowing the sign of the coefficient under investigation. We are also using a cut-off of 95%, functionally equivalent to an alpha of 0.05, when interpreting whether effects are statistically relevant.

Our motivation for using Bayesian analysis is twofold. First a Bayesian framework allows more intuitive summary statistics and more rigorous analysis of effects by using a posterior distribution of an effect, rather than a point estimate of an effect. Second, a Bayesian approach allows us to transparently include previous findings in our model. In other words, our observed data can be presented as a function of the data that came before in previous studies. A number of good textbooks exist that can further elaborate on these advantages, but we recommend ‘Doing Bayesian Data Analysis’ by John Kruschke^24^.

### Construction of priors

Priors were based on data from a longitudinal study demonstrating a change in authoritarian subfactors within the same individuals at two timepoints, 7 months apart^4^. All priors described below are also summarised adjacent to results of the regression analyses.

#### Correlations between pre- and post-ACT scores

Having access to the full data of this previous study, we were able to correlate authoritarian subfactors between the two timepoints which yielded correlations of approximately 0.80 across all three subfactors (Table 2). We chose to use a normal distribution for the prior and apply the same mean value for the lagged regressors of ACT subfactors, 0.80, and used an SD of 0.10, reflecting uncertainty due to a different sampled country, and study design.

**Table 2:** Pearson correlation between initial and follow-up ACT measures using the sample reported in Winter et al.^2,4^.

Subfactor	Estimate	95% CI	
		Lower	Upper
Submission	0.79	0.77	0.82
Conventionalism	0.88	0.86	0.89
Aggression	0.87	0.86	0.89

#### Effects of primes

The multilevel regression analysis conducted in Winter et al.^2,4^ used time in a non-experimental developmental study as a predictor of change in ACT subfactors between national Alert Levels in New Zealand. In the present study we embraced the assumption that the influence of terror and COVID-19 primes relative to the control would be analogous to the effect of national Alert Levels on perceived threat. We used the normal distribution parameterised with a mean of 0.05 for conventionalism and aggression, then 0.10 for submission for both COVID-19 and terror conditions (the approximate coefficients yielded in Winter et al.^2,4^. We used an SD of 0.10, over double the uncertainty observed by Winter et al.^2,4^, across all three subfactors and both prime conditions. Inflating the SD reflected our uncertainty about the sample in another country, and an effect being instigated on a much shorter time scale. All priors also encompassed zero, allowing a probability that no effect would be identified in the data.

#### Effects of covariates

We expected pre-exposure (baseline) ACT subscale scores to account for most individual differences which would naturally control for age and sex implicitly. Therefore, we did not expect much chance of significant covariates. Where an effect might exist, we expected it to be very small. We therefore used normal distributions with means of zero for age, sex, and political beliefs. However, we varied the SD for each measure, reflecting the breadth of plausible values in each case. Sex only had two levels, male or female, and thus the respective coefficient could vary over a larger range than age or the seven-point Likert scale for political orientation. Thus, we set the SD for sex to 0.50, the SD for political beliefs to 0.10, and the SD for age to 0.05. As a reference on what size of effect this allows, the SD of each ACT subfactor in our present data ranged from 1.41 to 1.62, so these priors allow quite large changes in score to be credible. These priors essentially provided moderate regularisation but did little to inform the model on the probability of an effect.

## Results

In this section we present the fit statistics for the latent variable structures generated using CFA. The effect of the prime manipulation is investigated using the two measures of threat. Lastly, and central to the study, we investigate whether there was a change in people’s levels of the three authoritarian dimensions based on their exposure to a specific prime. Descriptive differences between the conditions can be seen in Table 3.

**Table 3:** Mean and standard deviation for each authoritarian dimension pre- and post-prime for each of the three conditions (standard deviation in parentheses).

Condition	Aggression		Submission		Conventionalism	
	Pre	Post	Pre	Post	Pre	Post
Control	− 0.03 (1.56)	− 0.03 (1.41)	− 0.08 (1.66)	− 0.12 (1.49)	− 0.02 (1.66)	− 0.07 (1.62)
COVID-19	0.26 (1.56)	0.2 (1.4)	0.28 (1.48)	0.23 (1.41)	0.14 (1.61)	0.11 (1.52)
Terrorism	− 0.25 (1.55)	− 0.15 (1.42)	− 0.21 (1.54)	− 0.13 (1.49)	− 0.15 (1.67)	− 0.07 (1.71)

Note that all numbers are near zero due to CFA producing latent variables on a standardized scale centred on zero.

### Effect of primes

#### COVID-19 threat

**Figure 1:**
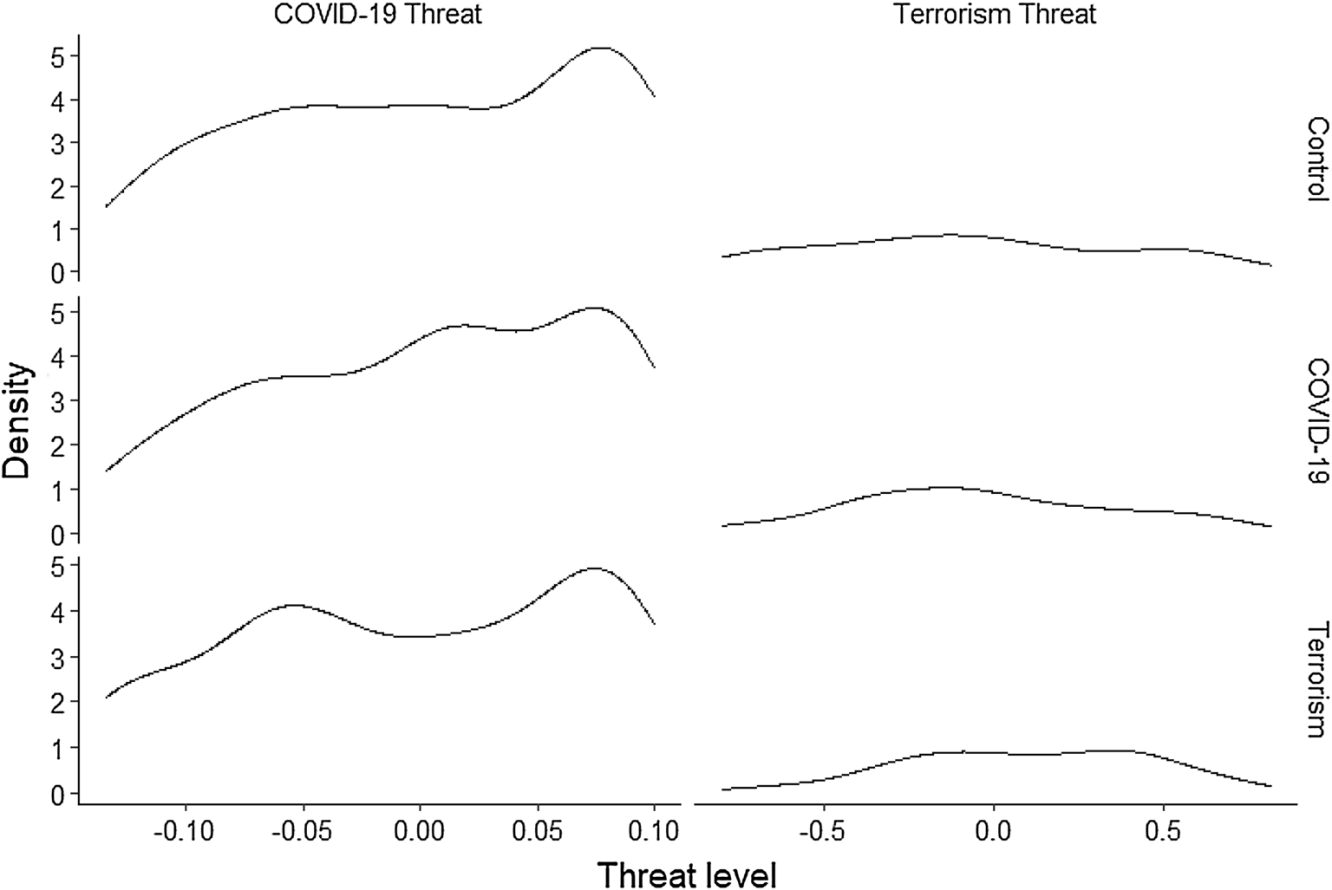
Density plots of the two threat measures by each prime condition. COVID-19 threat demonstrated a ceiling effect, whereas terrorism threat yielded a spread of values across its entire range.

Inconsistent with predictions, we found no effect of the COVID-19 prime on COVID-19 threat, but there was a 95% posterior probability that the terror prime had a negative effect on the perception of COVID-19 threat (Table 4, Fig. 1). The lack of effect of the COVID-19 prime may be explained by an apparent ceiling effect (i.e., everyone was worried about the threat of COVID-19 and the manipulation could not increase that worry further). Participants are living every day with a constant reminder of COVID-19 as a very real threat in their lives. The effect of the terror prime was not hypothesised despite having a reasonably high probability of reducing COVID-19 threat relative to threat levels observed in the control group. This may be because introducing a terrorism threat competes for attention with the everyday threat of COVID-19. Alternatively, it is worth noting that the terrorism prime dismissed COVID-19 as a prominent threat and this framing may have elicited a reduction independently of the elicitation of terrorism threat.

**Table 4:** Results of multivariate regression analyses showing between-subjects effects of each prime condition.

Outcome	Predictor	Estimate	Error	95% CI		pp
				Lower	Upper	
Covid threat	Intercept	− 0.05	0.02	− 0.09	− 0.02	
	Covid prime	0.00	0.01	− 0.02	0.02	0.51
	Terror prime	− 0.01	0.01	− 0.03	0.00	0.95
	Age	0.00	0.00	0.00	0.00	0.79
	Sex (male)	− 0.01	0.01	− 0.02	0.01	0.86
	Political beliefs	0.02	0.00	0.01	0.02	1.00
Terror threat	Intercept	0.09	0.12	− 0.14	0.31	
	Covid prime	0.05	0.06	− 0.06	0.17	0.82
	Terror prime	0.17	0.05	0.06	0.27	1.00
	Age	0.00	0.00	0.00	0.00	0.54
	Sex (male)	− 0.09	0.05	− 0.19	0.00	0.97
	Political beliefs	− 0.02	0.01	− 0.05	0.00	0.96

Credible intervals (CI) denote the 2.5% and 97.5% quantiles of the posterior distribution. Posterior probability (pp) denotes the proportion of posterior samples above or below zero depending on direction of the effect.

#### Terrorism threat

Individuals exposed to the terror prime yielded significantly higher levels on the terror threat scale relative to individuals in the control prime, i.e., there is strong evidence that the terror prime effectively induced a perception of threat (Table 4). There was no effect of the COVID-19 prime on levels of terrorism threat. Of note, the terrorism threat scale was the only scale that did not meet the predetermined thresholds for fit statistics with an RMSEA of 0.2 and CFI of 0.8. In spite of unideal fit statistics we supported the influence of the terrorism prime with this scale. As it was not used in our primary analyses, we had no further concern over the fit statistics.

### Change in authoritarianism self-reports

**Figure 2:**
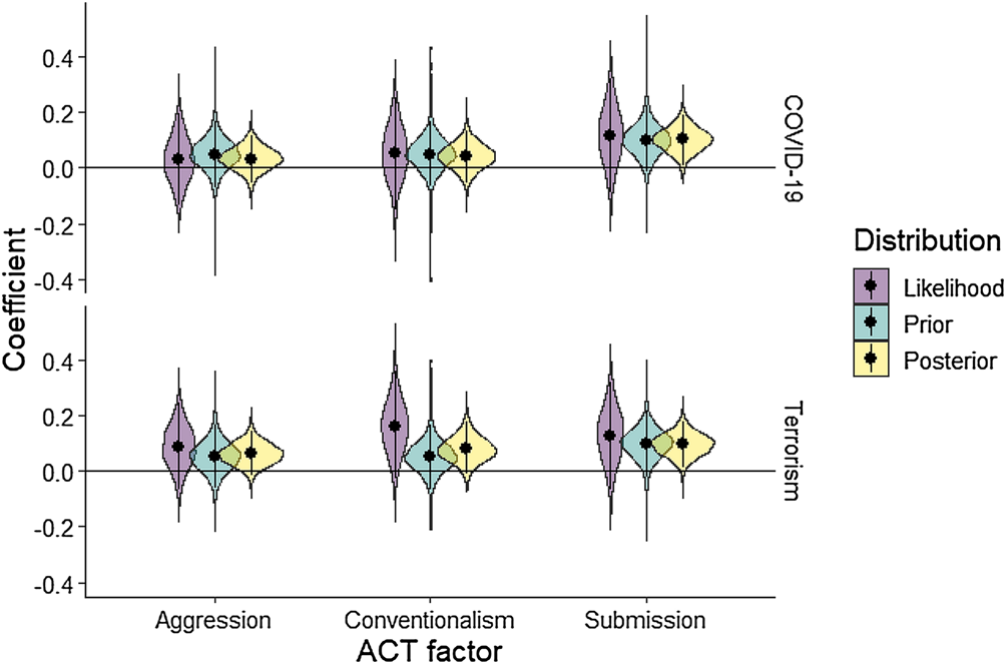
Likelihood, prior, and posterior distributions of posterior samples for each prime condition relative to the control prime. The black dot marks the mean, and tails within each shape denote the 95% CI based on the ETI defined above. Horizontal line gives reference to zero, implying no effect of the prime with each authoritarian factor.

Priors had a moderate impact on the posterior distribution of each effect (Fig. 2). Specifically in the case of COVID-19 and terrorism primes, Fig. 2 shows that the likelihood tended to manifest greater uncertainty than the prior, yet all priors and likelihood distributions did show similar patterns. When assessing the posterior probabilities, the COVID-19 prime yielded an 86% probability of an effect above zero with a non-informative analysis, and 99% probability with informed priors (Table 5). Similarly, our terrorism prime sat at a 90% probability of an effect above zero with non-informative analysis, rising to 98% probability with informed priors. These results suggest that both primes boosted an increase in submission scores from pre-test to post-test.

**Table 5:** Results of multivariate regression analysis predicting post prime authoritarian factors.

Outcome	Predictor	Estimate	Error	95% CI		pp	
				Lower	Upper	Default	Informed
Submission_Post_	COVID-19 prime	0.10	0.05	0.01	0.19	0.86	0.99
	Terrorism prime	0.09	0.04	0.01	0.18	0.90	0.98
	Political beliefs	− 0.08	0.04	− 0.16	− 0.01	1.00	0.98
	Age	− 0.05	0.04	− 0.13	0.02	0.96	0.93
	Sex (male)	− 0.02	0.06	− 0.15	0.10	0.59	0.63
	Submisssion_Pre_	0.75	0.02	0.71	0.80	1.00	1.00
Aggression_Post_	COVID-19 prime	0.03	0.04	− 0.05	0.12	0.63	0.80
	Terrorism prime	0.06	0.04	− 0.02	0.14	0.87	0.94
	Political beliefs	− 0.08	0.04	− 0.15	− 0.01	1.00	0.98
	Age	− 0.05	0.03	− 0.11	0.01	0.96	0.95
	Sex (male)	0.02	0.05	− 0.09	0.12	0.65	0.61
	Aggression_Pre_	0.78	0.02	0.73	0.83	1.00	1.00
Conventionalism_Post_	COVID-19 prime	0.04	0.05	− 0.05	0.13	0.70	0.83
	Terrorism prime	0.08	0.05	− 0.01	0.18	0.95	0.96
	Political beliefs	− 0.09	0.04	− 0.18	0.00	1.00	0.98
	Age	− 0.01	0.04	− 0.08	0.07	0.57	0.57
	Sex (male)	0.11	0.07	− 0.01	0.24	0.98	0.96
	Conventionalism_Pre_	0.84	0.03	0.78	0.89	1.00	1.00

95% CI is the credible interval derived by trimming 2.5% off each end of the distribution, i.e., the Equal Tailed Interval (ETI). Two posterior probabilities (pp) are presented for each measure, one for default priors and one for informed priors.

The aggression subfactor did not appear to yield a reliable effect with posterior probabilities as both prime conditions encompassed zero as a credible value even with informed priors (yielding an 80% posterior probability above zero with COVID-19 and 94% probability with terrorism). Conventionalism, however, did yield a high probability both with non-informed and informed analyses having a 95% posterior probability for the terrorism prime, but not the COVID-19 prime. This result implies that the terrorism prime increased conventionalism scores from pre-test to post-test, but the COVID-19 prime resulted in no change across conventionalism scores.

It should be noted that in all cases, political beliefs yielded a reliable effect across all three factors, in that more liberal people reported lower levels of change in their authoritarian scores. In other words, conservative people exhibited a general increase in all three ACT subfactors from pre-test to post-test. We also noted lower levels of submission and aggression as age increased, but no relationship between age and conventionalism was found. In contrast, there was no difference between males and females for submission and aggression, but males tended to report higher levels of conventionalism than females.

Some readers less familiar with Bayesian analysis may wish to consider comparisons with the equivalent frequentist analysis. In this instance, one minus the posterior probability (1-pp), can be considered equivalent to the p-value that would be yielded by a frequentist analysis (Table 5). This is because the default prior is merely the observed likelihood, i.e., the probability of the data given a hypothesis. Similarly, you may wish to consider the credible interval of the likelihood in Fig. 2 as similar to a frequentist confidence interval, and the posterior is the combined probability of what we expected from previous research (prior) and the likelihood. We do caveat, however, that there are exceptions to this rule and conceptual differences exist, but this thinking may aide those less familiar with Bayesian analysis.

## Discussion

The aim of the present study was to utilise threatening primes to manipulate levels of specific dimensions of authoritarianism. Testing of our experimental manipulation check suggested that the COVID-19 prime failed to yield a meaningful difference relative to participants who experienced the neutral control prime. The domestic terrorism prime, by contrast, yielded a meaningful increase in level of threat relative to the control prime and, in contrast, may have led to a reduction in perceived COVID-19 threat (as all three groups completed both terrorism and COVID-19 threat scales). Despite mixed results from our manipulation check, participants who experienced the COVID-19 or terrorism primes reported higher levels of authoritarian submission after the prime compared to before the prime, relative to those who experienced the neutral control prime. It is unlikely that the experimental primes exerted any effect on authoritarian aggression, but there did seem to be an indication that authoritarian conventionalism rose for those individuals who experienced the terrorism prime relative to the control prime. Notably, it seems that perceived threats of COVID-19 and terrorism led to an increase in authoritarian submission, supporting our first hypothesis. Thus, we have successfully replicated the longitudinal effect of submission change aligned with COVID-19 noted by Winter et al.^2^ in their non-experimental study, and we extended the literature on experimental studies of authoritarianism that had previously relied on a unidimensional measure of authoritarianism.

### Authoritarian submission fluctuates in response to threat

In alignment with other research, the change we demonstrated in submission can be interpreted as a situational threat influencing beliefs that the world is a dangerous place and inducing motivation for collective security^14,25,26^. In this specific context, we would speculate that the threats bolstered world views of dissension and disagreement within society that needed to be addressed by greater social order and conformity. Conventionalism, by contrast, is driven more by moral uncertainty and is thought to be addressed by strict adherence to traditional norms^14^. It may then make sense that we observed a reasonable probability that conventionalism increased due to the terrorism prime but not the COVID-19 prime. At the time of the study, there were many norms and rules in place to address the risks of COVID-19, but restrictions limiting the threat of domestic terrorism and protecting the rules and order of the in-group may be less salient.

There was a 94% probability that the terrorism prime was associated with an increase in authoritarian aggression, which we did not hypothesise. Aggression is theorised to arise from direct physical threats, and authoritarian aggression manifests as an attitude of punitive and coercive social control directed toward people who fail to conform. In the terrorism prime, we did not clearly distinguish between direct (personal) and indirect (societal) domestic terrorism threats, which may have led to the current findings. Our prediction aligned conceptually with research using a unidimensional measure of authoritarianism indicating a bigger difference in a group that experienced a direct personal threat from terrorism relative to an indirect collective threat from terrorism^12^. One potential explanation for this discrepancy between the terrorism and COVID-19 primes in the present study is that the terrorism prime was interpreted as a direct personal threat, more so than the COVID-19 prime. The addition of a measure for collective threat and personal threat with a similar paradigm as that utilised herein, would be sufficient to test this potential explanation.

Although authoritarianism has been conceptualised as three distinct but related attitudinal clusters for some time, research still tends to bundle the three attitudinal clusters together, a limitation that should be more strongly considered in ongoing research. In the present study we saw distinct differences between submission relative to aggression and conventionalism, a nuance that could help explain the often conflicting results in the literature.

## Limitations and future directions

A limitation in this study was the failure for the COVID-19 prime to pass a manipulation check (although it did still show an experimental effect on authoritarianism dimensions). One innocuous explanation is that the COVID-19 threat scale we drew from the literature was not a sufficiently sensitive scale. Another, perhaps more meaningful explanation, is that participants were already primed with some level of threat due to chronic awareness and fear of COVID-19, and this ambient perception may have diminished the magnitude of threat that could be induced by increasing the already high level of salience. Considering the distributions of COVID-19 threat across the three groups, it seems that both these options are likely. The distributions contained a significant left skew across all three groups.

High COVID-19 threat across all participants prior to the study is consistent with longitudinal studies of authoritarianism and COVID-19 threat, whereby a New Zealand sample^4^ reported higher levels of authoritarian submission when the COVID-19 restrictions were highest, compared to when COVID-19 had been eliminated in their society. The present sample was tested during a stable, low threat time in terms of infected cases and restrictions in the United States. When designing the present study, we relied heavily on New Zealand data which implied the perceived threat of COVID-19 was very low and we carried that assumption through to the US sample. However, it may have been that the environment in the United States still induced a reasonable perception of COVID-19 threat relative to the COVID-19-free environment from New Zealand. This difference would mean that participants already had elevated levels of COVID-19 threat regardless of which prime condition they received, and any potential effects would have been diminished.

Certainly, the interpretation of different perceptions of COVID-19 risk across countries gives interesting avenues for future research concerning cross-cultural and international comparisons. Understanding similar threats in different environments could be used to understand whether the extent of change in authoritarian dimensions is comparable across different cultures. As we have highlighted in the present study, baseline levels of authoritarianism are a moderator to any reactions to perceived threat. Therefore, the response to a perception of COVID-19 threat may differ significantly between a country such as New Zealand that had a left-wing leadership and political support of scientific leaders, relative to the United States that initially had a right-wing leadership sowing doubt in the scientific leaders of the day.

The perceived COVID-19 threat seems to have been lower in the terrorism prime group compared to the control group. This effect was far smaller than the effect we observed in terrorism threat for the terrorism prime group, but its high probability should not be dismissed. We would tentatively interpret this effect as a decrease in the attention granted to COVID-19 as a societal threat when posed with a competing societal threat. That is, the participants were potentially (and perhaps temporarily) distracted by the perception of a terrorist threat which reduced their concerns of COVID-19. In future work, one consideration could be presenting a series of primes and retesting to see if successive societal threats can diminish previously evoked perceptions of an unrelated societal threat, and then re-emerge later as threats fade or are reinstated. Notwithstanding this avenue of research, the very small effect of the COVID-19 prime, assuming it was undetectably small for our scale of COVID-19 threat, may have arisen due to a lack of perception for personal threat^12,13^. The role of personal versus a collective threat will also need to be considered in these future studies, but unfortunately cannot be isolated in the present study.

A further element that could be explored is that of political orientation. In the present study we found that liberals unsurprisingly had lower mean levels of each authoritarian dimension relative to conservatives. In New Zealand samples, different responses to threat based on political orientation have been identified in the context of COVID-19^2^. In our study we had planned our sample size and allocation of experimental conditions without consideration of more in depth analysis on political orientation. In future research, we would encourage the study of political orientation as a moderator to determine what, if any, it may have on the magnitude of change in authoritarian dimensions when participants are faced with a threatening prime. Indeed, it could have been the case that in the present study, a subgroup of participants responded to the threat prime and/or altered their authoritarian beliefs, such as those who are politically conservative.

## Conclusions

In conclusion, the present study demonstrated that two forms of societal threat, COVID-19 and terrorism, can elicit changes in authoritarian submission and conventionalism under specific conditions. The differences we observed were on a rapid time scale in the order of minutes. In contrast with previous research that only highlighted longitudinal effects on COVID-19, the present study also contrasted findings with the threat of domestic terrorism. Highlighting that both threats demonstrated similar results and that it is likely findings can be generalised across multiple kinds of threat. We therefore conclude that we have support for the view that authoritarianism facets contain a state-like component and should be treated as a multidimensional structure of attitudes that can fluctuate quickly in response to the perception of a societal threat. This sits well with the motivational model of authoritarianism put forward by Duckitt et al.^6^, that suggests that in the face of a societal threat, individuals are motivated to adopt authoritarian beliefs. The adoption of such beliefs enhances social cohesion which is thought to help protect an in-group from an external threat. We found that authoritarian submission fluctuated most readily among the three subfactors. These experimental findings also align with research suggesting that submission tends to change the most in the face of COVID-19 threat^2,4^. These findings highlight the need for research to consider the multidimensional nature of authoritarianism in their investigations rather than treating authoritarianism as a unidimensional structure.

### Supplementary Information

Supplementary Information.

## References

[1] Schnelle C, Baier D, Hadjar A, Boehnke K (2021). Authoritarianism beyond disposition: A literature review of research on contextual antecedents. Front. Psychol..

[2] Winter T, Jose PE, Riordan BC, Bizumic B, Ruffman T, Hunter JA, Hartman TK, Scarf D (2022). Left-wing support of authoritarian submission to protect against societal threat. PLoS ONE.

[3] Russo S, Roccato M, Merlone U (2020). Actual threat, perceived threat, and authoritarianism: An experimental study. Spanish J. Psychol..

[4] Winter T, Riordan BC, Bizumic B, Hunter J, Jose PE, Duckitt J, Scarf D (2022). Longitudinal change in authoritarian factors as explained by political beliefs and a distrust of science. Front. Polit. Sci..

[5] Altemeyer B (1996). The Authoritarian Specter.

[6] Duckitt J, Bizumic B, Krauss SW, Heled E (2010). A tripartite approach to right-wing authoritarianism: The authoritarianism-conservatism-traditionalism model. Polit. Psychol..

[7] Dunwoody PT, Funke F (2016). The aggression-submission-conventionalism scale: Testing a new three factor measure of authoritarianism. J. Soc. Polit. Psychol..

[8] Adorno, T., Frenkel-Brunswik, E., Levinson, D. J., & Sanford, R. N. *The Authoritarian Personality* (First). (Harper & Brothers, 1950).

[9] Altemeyer B (1981). Right-Wing Authoritarianism.

[10] Funke F (2005). The dimensionality of right-wing authoritarianism: Lessons from the dilemma between theory and measurement. Polit. Psychol..

[11] Peterson BE, Pratt MW, Olsen JR, Alisat S (2016). The authoritarian personality in emerging adulthood: Longitudinal analysis using standardized scales, observer ratings, and content coding of the life story. J. Pers..

[12] Asbrock F, Fritsche I (2013). Authoritarian reactions to terrorist threat: Who is being threatened, the Me or the We?. Int. J. Psychol..

[13] Feldman S, Stenner K (1997). Perceived threat and authoritarianism. Polit. Psychol..

[14] Jugert P, Duckitt J (2009). A motivational model of authoritarianism: Integrating personal and situational determinants. Polit. Psychol..

[15] Little, T. D. & Rhemtulla, M. Planned missing data designs for developmental researchers. *Child Dev. Perspect.* **7**(4), 199–204. 10.1111/cdep.12043 (2013).

[16] Rhemtulla, M. & Little, T. D. Planned missing data designs for research in cognitive development. *J. Cogn. Dev.* **13**(4), 425–438. 10.1080/15248372.2012.717340 (2012). 10.1080/15248372.2012.717340 PMC3857527 24348099

[17] Kachanoff FJ, Bigman YE, Kapsaskis K, Gray K (2021). Measuring realistic and symbolic threats of COVID-19 and their unique impacts on well-being and adherence to public health behaviors. Soc. Psychol. Pers. Sci..

[18] Sinclair SJ, LoCicero A (2007). Fearing future terrorism: Development, validation, and psychometric testing of the terrorism catastrophizing scale (TCS). Traumatology.

[19] van Buuren S, Groothuis-Oudshoorn K (2011). mice: Multivariate imputation by chained equations in R. J. Stat. Softw..

[20] R Core Team. *R: A Language and Environment for Statistical Computing*. (R Foundation for Statistical Computing, 2021) https://www.R-project.org/.

[21] Azur MJ, Stuart EA, Frangakis C, Leaf PJ (2011). Multiple imputation by chained equations: What is it and how does it work?. Int. J. Methods Psychiatr. Res..

[22] Rosseel Y (2012). lavaan: An R package for structural equation modeling. J. Stat. Softw..

[23] Jorgensen, T. D., Pornprasertmanit, S., Schoemann, A. M., & Rosseel, Y. *semTools: Useful Tools for Structural Equation Modeling*. https://CRAN.R-project.org/package=semTools (2021).

[24] Kruschke, J. K. Doing Bayesian data analysis. In *Doing Bayesian Data Analysis*, 193–219 (Elsevier, 2015) 10.1016/b978-0-12-405888-0.00008-8.

[25] Duckitt J, Bizumic B (2013). Multidimensionality of right-wing authoritarian attitudes: Authoritarianism-conservatism-traditionalism. Polit. Psychol..

[26] Duckitt J, Fisher K (2003). The impact of social threat on worldview and ideological attitudes. Polit. Psychol..

