# Supplementary material for: Experimentally induced changes in authoritarian submission as a response to threat
Source: Sci Rep. 2023 Oct 31;13:18699. doi: 10.1038/s41598-023-44713-3 (PMC10618266; doi:10.1038/s41598-023-44713-3)
Supplement: Supplementary file 1 — Supplementary Information. [file 41598_2023_44713_MOESM1_ESM.docx]

**Appendix A**

News clipping collage for COVID-19 and domestic terrorism primes

**Figure s1.** News clipping collage for COVID-19 prim

**
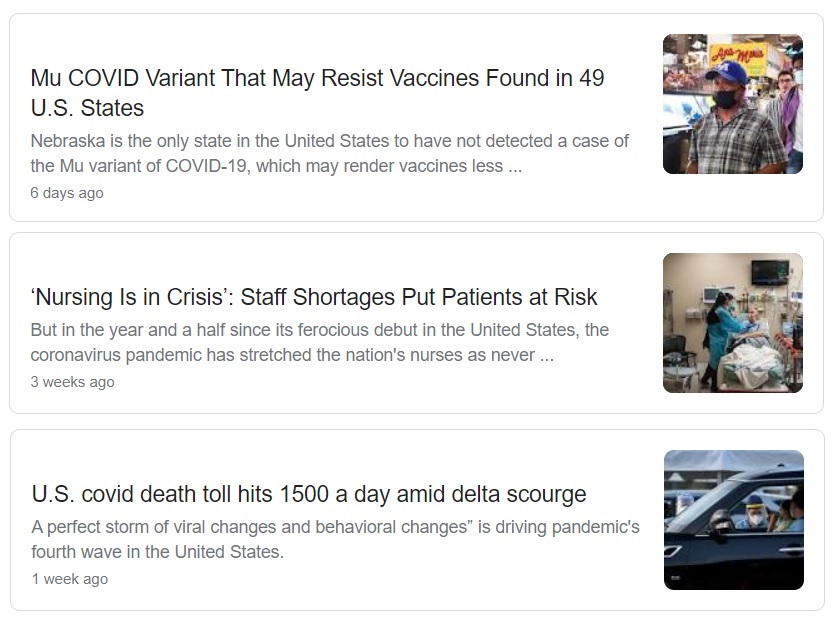
**

**Figure s2.** News clipping collage for domestic terrorism prime.


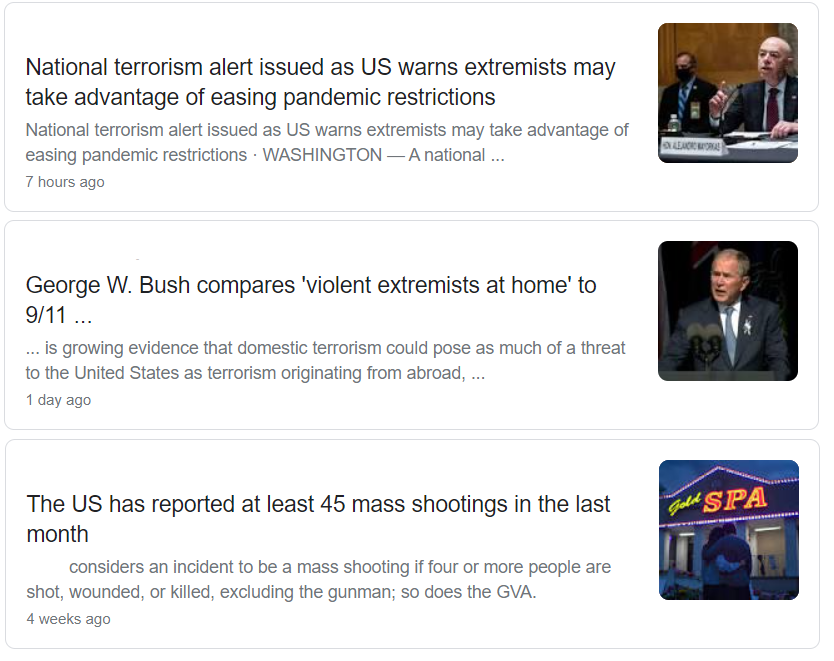


**Appendix B**

TerrorismCatastrophizing Scale (TCS)

The scale consists of 9 items and participants responded using a 5-point Likert scale ranging from 1 (strongly agree) to 5 (strongly disagree).

1. There is little I can do to protect myself from terrorism
2. I frequently think about the threat of future terrorism
3. The threat of terrorism does not enter my mind that often
4. I worry that terrorism will only get worse as time passes
5. I worry that the threat of terrorism will never end
6. I often dwell on the threat of future terrorism
7. I believe the future is dark with respect to the threat of terrorism
8. I have a lot of power in keeping myself safe from terrorism
9. I lack control in defending myself and my loved ones against terrorism
